# Stable Heterogeneity for the Production of Diffusible Factors in Cell Populations

**DOI:** 10.1371/journal.pone.0108526

**Published:** 2014-09-30

**Authors:** Marco Archetti

**Affiliations:** School of Biological Sciences, University of East Anglia, Norwich, United Kingdom; University of Sheffield, United Kingdom

## Abstract

The production of diffusible molecules that promote survival and growth is common in bacterial and eukaryotic cell populations, and can be considered a form of cooperation between cells. While evolutionary game theory shows that producers and non-producers can coexist in well-mixed populations, there is no consensus on the possibility of a stable polymorphism in spatially structured populations where the effect of the diffusible molecule extends beyond one-step neighbours. I study the dynamics of biological public goods using an evolutionary game on a lattice, taking into account two assumptions that have not been considered simultaneously in existing models: that the benefit of the diffusible molecule is a non-linear function of its concentration, and that the molecule diffuses according to a decreasing gradient. Stable coexistence of producers and non-producers is observed when the benefit of the molecule is a sigmoid function of its concentration, while strictly diminishing returns lead to coexistence only for very specific parameters and linear benefits never lead to coexistence. The shape of the diffusion gradient is largely irrelevant and can be approximated by a step function. Since the effect of a biological molecule is generally a sigmoid function of its concentration (as described by the Hill equation), linear benefits or strictly diminishing returns are not an appropriate approximations for the study of biological public goods. A stable polymorphism of producers and non-producers is in line with the predictions of evolutionary game theory and likely to be common in cell populations.

## Introduction

Cooperation for the production of diffusible molecules is commonly observed in cell populations, from bacteria to eukaryotes [1]: bacteria, for example, produce molecules that contribute to population growth (like pyocyanin [2] and pyoverdine [3]), that enable the buildup of biofilms [4] or that confer resistance to antibiotics [5]; yeast cells produce invertase that catalyzes the hydrolysis of sucrose [6], and cancer cells produce growth factors that contribute to tumour expansion [7]. Because the effect of diffusible molecules is not limited to the producer cells, a mutant cell not producing the molecule can still benefit from the presence of its neighbour producers. The free-rider advantage enjoyed by non-producer cells may lead to an increase in their frequency in the population and drive producers to extinction, with a consequent reduction in average fitness for the population - similar to what is often referred to as “the tragedy of the commons” [8]. It is understood, however, that because this free-rider advantage is frequency-dependent, if the benefit conferred by the public good is non-linear, the dynamics is generally more complex and in well-mixed populations it can lead to a stable coexistence of producers and non-producers [9]. Whether this is also the case in spatially structured populations, however, is unclear.

In the study of public goods games in spatially structured populations it is usually assumed [10] that an individual’s action affects only the fitness of individuals one node away and that an individual’s fitness is the sum of all the payoffs accumulated in all the groups she belongs to (all the groups formed by the one-step neighbours of her one-step neighbours). This is reasonable for interactions in human social networks, but not for cellular networks, in which molecules typically diffuse beyond a cell’s one-step neighbours, and in which the benefit for a cell is a function of the number of producer cells within the diffusion range of the molecule. In order to study diffusible public goods, therefore, one must decouple the interaction neighbourhood (the group playing the game, defined by the diffusion range of the molecule) and the update neighbourhood (the one-step neighbours). While such models have been used to study a simple two-person game with a linear benefit function (the prisoner’s dilemma) on a regular lattice [11], [12] only recently it has been used to study the dynamics of multi-player public goods games (which are appropriate for the study of biological molecules) and there seems to be no consensus on the conclusions of these studies.

Borenstein et al. [13] showed that in a 2-D model with diffusion and linear benefits producers and non-producers can never coexist. Scheuring [14] showed, instead, stable coexistence in a 1-D model with concave benefits (diminishing returns) and even (although rarely) with linear benefits, depending on the initial conditions of the system. Archetti [15] showed that coexistence is the typical outcome of the dynamics in a 2-D model with diffusion, but did not take into account the fact that the efficacy of the diffusible molecule may decline with the distance form the source. Allen et al. [16] studied a model with diffusion and linear benefits, but did not investigate the possibility of coexistence of the two types, since in their finite stochastic population one of the strategies eventually goes to fixation (it is known, however, that in the presence of a stochastically stable polymorphism, coexistence in large populations is possible since fixation time increases exponentially with population size [17]).

A number of different assumptions in these studies [13]–[16] can account for the different conclusions about the possibility of a stable polymorphism. I will analyse two assumptions that seem the most prominent differences in the 2-D models described above: the shape of the diffusion gradient of the molecule (the efficacy of the molecule as a function of the distance from the producer cell) and the shape of the benefit function (the amount of public good produced as a function of the fraction of producers). In Borenstein et al. [13], Allen et al. [16] and Scheuring [14] the diffusion gradient is a smooth decreasing function, whereas in Archetti [15] the diffusion gradient is a step function. In Borenstein et al. [13] and Allen et al. [16] the benefit function is linear, whereas Scheuring [14] uses both linear and concave benefits, and Archetti [15] uses a variety of shapes, including linear, concave and sigmoid benefits. It is possible that the lack of a stable polymorphism reported by Borenstein et al. [13] is due to the fact that the benefit used in their model is linear, or it is possible that the stable polymorphism observed by Archetti [15] is due to the fact that the diffusion gradient in his model is a step function. Scheuring [14] showed that concave benefits can lead to coexistence, a result that is in contrast with both Borenstein et al. [13] and Archetti [15] but is not necessarily applicable to 2-D models and sigmoid benefit functions.

I will use a 2-D model that takes into account a variety of non-liner benefits (not analysed by Borenstein et al. [13]) and of smooth diffusion gradients (not used by Archetti [15]), extending therefore to 2-D and sigmoid benefits the results obtained by Scheuring [14] for linear and concave benefits in a 1-D model. The results of this extended model will help clarify what assumptions (diffusion gradient or non-linear benefits) are essential for an analysis of the problem, and whether a stable polymorphism is indeed possible with public goods involving diffusible biological molecules.

## The model

### Topology

Individual cells occupy all individual nodes of a planar graph, a regular 30×30 square lattice in which each node has four neighbours (Von Neumann neighbourhood) and the opposing edges are connected to form a toroidal network, in order to avoid edge effects. Differently from the standard approach (in which an individual’s group is limited to her one-step neighbours and an individual plays multiple games centred on each of her neighbours [10]), here the interaction neighbourhood and the update neighbourhood are decoupled (the approach used by Archetti [15] and by Borenstein et al. [13]): a cell’s group (of size *n*) is not limited to her one-step neighbours but is defined by the diffusion range *D* of the molecule. Group size for a cell is given by the number of cells within *D* nodes from that cell; in the Von Neumann neighbourhood, *n* = 2*D*(*D*+1)+1.

While in a model with a fixed diffusion range (a step function, in which the molecule is 100% effective up to a fixed distance from the source, and completely ineffective beyond that range) a cell’s payoff can be determined simply by the number of producer cells within a given range from that cell, in a model with a diffusion gradient each cell receives contributions from other producer cells within a diffusion range *D* of the molecule, each contribution weighted by the distance *i* (the number of nodes) from that cell according to the following function:
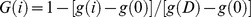
where



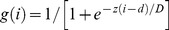



The relative value of *d* and *D* determines the shape of the diffusion gradient, which is always decreasing but can be concave (*d* = *D*), convex (*d<<D*) or sigmoid (intermediate values of *d<D*). For example, if *d = D*/2 and there are six producer cells all *d* nodes away from a focal cell (*i = d* for all producers), *G*(*d*) = 1/2, that is, only half of each producer’s contribution is available for the focal cell, hence the *weighted* number of producers (the sum of all the contributions) in that focal cell’s group is three. The parameter *z* controls the steepness of the gradient at the inflection point: *z→*0 models a linear gradient; *z→∞* models a step function equivalent to the one used by Archetti [15].

### The game

We assume, as is standard, that there are two types of cells, producers and non-producers. Producers pay a cost *c* that non-producers do not pay (0<*c*<1). All cells (producers and non-producers) benefit from the public good produced by all the cells in their group. The benefit function is.
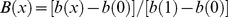
where



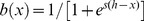
is a function of the weighted fraction of producers *x* in the group that is, the weighted number of producers *j* in the group divided by group size *n* (see “*Topology*”). The parameter *h* controls the position of the inflection point (*h→*1 gives strictly increasing returns and *h→*0 strictly diminishing returns) and the parameter *s* controls the steepness of the function at the inflection point (*s→∞* models a steep sigmoid function that is essentially an on/off switch; *s→*0 models linear benefits) [18]. *B*(*j*) is a simple normalisation of the logistic function *b*(*j*) (without the normalisation, low values of *s* would yield constant benefits rather than linear increasing benefits). While input-output functions in biochemistry are often described by the Hill equation [19], [20], we use this normalised logistic function because it enables to model not only sigmoid benefits but also linear and concave benefits, which are used by Scheuring [14] and Archetti [15], discussed by Borenstein [13], and used in models of public goods games in well-mixed populations [9].

A cell’s payoff is a function of the amount of factor produced by the group she belongs to. I use a birth-death process equivalent to the one used by Archetti [15] and by Borenstein et al. [13]. The process starts with a number of non-producer cells placed on the graph; at each round a cell *x* with a payoff P*_x_* is selected (at random) for update (death); a cell *y* (with a payoff P*_y_*) is then chosen among *x’*s neighbours. Two types of update are used: in the deterministic case, if P*_x_*>P*_y_*, no update occurs, while if P*_x_*<P*_y_*, *x* will adopt *y*’s strategy (unconditional imitation); in the stochastic case, replacement occurs with a probability given by (P_y_-P_x_)/M, where M ensures the proper normalization and is given by the maximum possible difference between the payoffs of *x* and *y* [10]. Results are obtained averaging the final 200 of 1000 generations per cell, averaged over 10 different runs.

## Results

### Sigmoid benefits lead to coexistence, with or without a diffusion gradient

**Figure 1:**
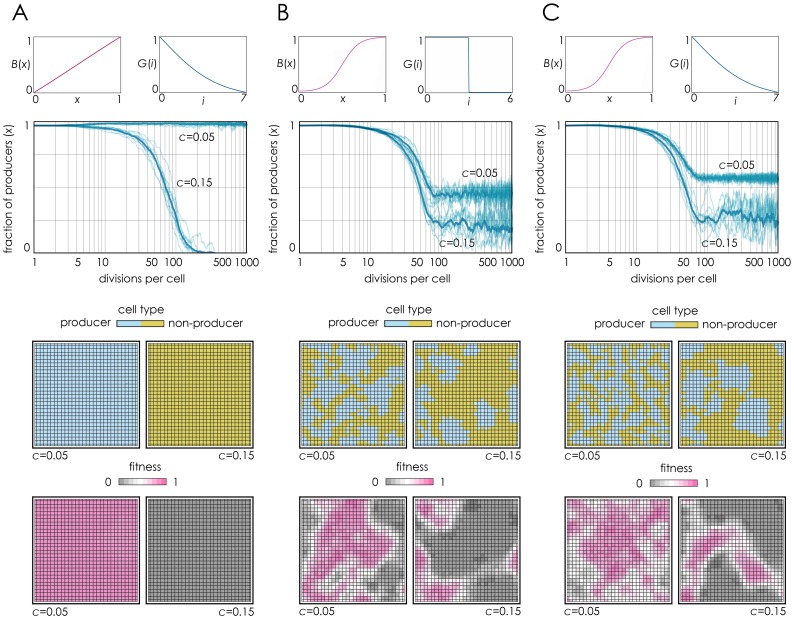
Realistic Hill coefficients lead to coexistence of producers and non-producers. For different benefit functions *B*(*x*) and gradients of diffusion *G*(*i*), the fraction of producers over time is show for *c* = 0.05 and *c* = 0.15. The lattices show the population after 1000 generations per cell. **A:** Linear benefit (*s* = 1, *h* = 0.5) with a diffusion gradient (*z* = 3, *d* = 0, *D* = 7). **B:** Sigmoid benefit (*s* = 20, *h* = 0.5) with no diffusion gradient (*z* = 1000, *d* = 3, *D* = 6). **C:** Sigmoid benefit (*s* = 20, *h* = 0.5) with a diffusion gradient (*z* = 3, *d* = 0, *D* = 7).

First, I checked that the results of two degenerate versions of the model correspond to the ones reported previously: with a linear benefit and a smooth diffusion gradient [13], no coexistence of producers and non-producers is observed [**Figure 1A**]; with no diffusion gradient and a non-linear benefit [15], producers and non-producers can coexist [**Figure 1B**]. These results therefore are in line with the two simpler models [13], [15] that led to opposing results. When benefits are non-linear, replacing the step function diffusion range [15] with a more realistic diffusion gradient [13] still leads to a coexistence of producers and non-producers [**Figure 1C**]. The shape of the benefit function, therefore, seems crucial for the outcome of the dynamics, whereas the diffusion gradient seems irrelevant.

### The result is robust and can hold even for concave benefits

**Figure 2:**
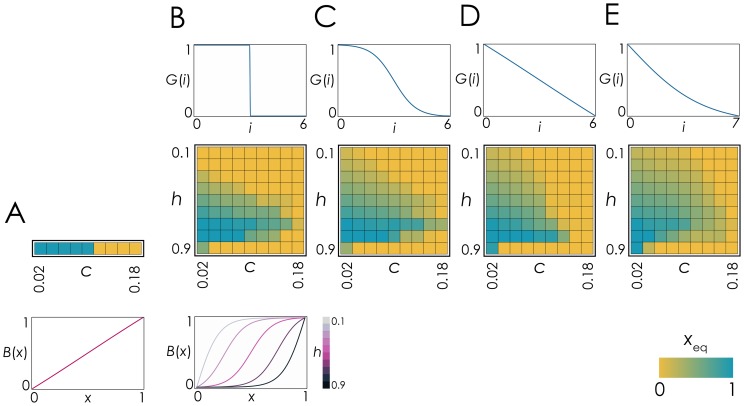
Different diffusion gradients allow coexistence of producers and non-producers. For different benefit functions *B*(*x*) and gradients of diffusion *G*(*i*), the contour plots show the fraction of producers at the stable mixed equilibrium (*x*_eq_) as a function of *h* (the inflection point of the benefit function) and *c* (the cost of producing the molecule). **A**: Linear benefit (*s* = 1, *h* = 0.5; any of the diffusion gradients in **B-E**). **B-E**: Sigmoid benefit (*s* = 20). **B**: Fixed diffusion range with no diffusion gradient (*d* = 3, *D* = 6, *z* = 1000). **C**: Sigmoid diffusion gradient (*d* = 3, *D* = 6, *z* = 10). **D**: Linear diffusion gradient (*d* = 3, *D* = 6, *z* = 1). **E**: Convex diffusion gradient (*d* = 0, *D* = 7, *z* = 3).

To check the robustness of the previous conclusion I analyse the dynamics under a variety of diffusion gradients and benefit function. While linear benefits always lead to the extinction of one of the two types [**Figure 2A**], as shown by Borenstein et al. [13], non-linear benefits can lead to coexistence under a wide range of parameters if the cost is not too high [**Figure 2B-E**], in line with previous results in well-mixed populations [9], and as shown by Archetti [15] for diffusible molecules in spatially structured populations. The type of diffusion gradient is largely irrelevant, whereas the shape of the benefit function is the major determinant of the dynamics.

While Borenstein et al. [13] analyse linear benefits, they suggest that concave benefits, like linear benefits, would not lead to coexistence. With diffusion gradients, however, concave benefits (diminishing returns) can actually lead to a stable polymorphism, even though under a much more limited parameter range than with sigmoid benefits [**Figure 2B–E**]. Therefore diffusion gradients are even more conductive to polymorphic equilibria than the simple diffusion range (step function) used by Archetti [15].

### No effect of diffusion gradients on population structure

**Figure 3:**
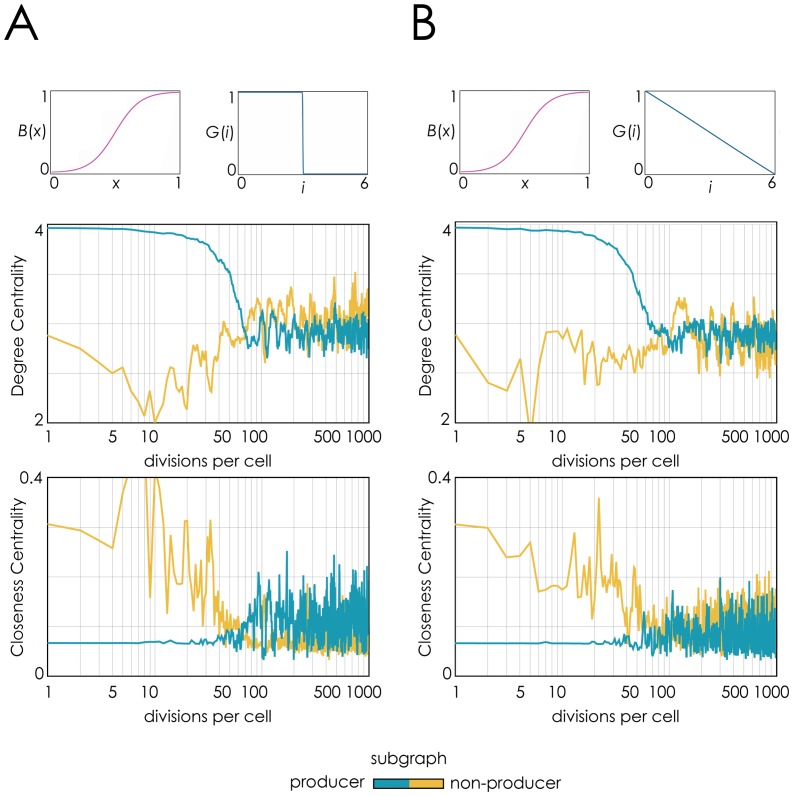
Diffusion gradients do not affect changes in population structure. Changes in degree centrality and closeness centrality over time are shown for the producer and non-producer subgraphs. **A**: Fixed diffusion range with no diffusion gradient (*d* = 3, *D* = 6, *z* = 1000). **B**: Linear diffusion gradient (*d* = 3, *D* = 6, *z* = 1).

Analysing changes in the topology of the subgraphs of producers and non-producers [**Figure 3**] reveals that the shape of the diffusion gradients does not affect significantly the spatial dynamics of the population. The degree centrality of the non-producer subgraph is only slightly lower (that is, non-producers have fewer neighbours in their clusters) when the molecule has a smooth decreasing diffusion gradient than when the diffusion is a step function; intermediate cases (not shown) lead to similar negligible differences. The closeness centrality (the inverse of the sum of the distance to all other vertices) is slightly higher for non producers and lower for producers (that is, non-producer clusters tend to be smaller and producer cluster bigger), but the differences are negligible. In short, whether diffusion is modelled as a gradient or not does not affect significantly the topology of the two subgraphs.

### Rationale of the results

**Figure 4:**
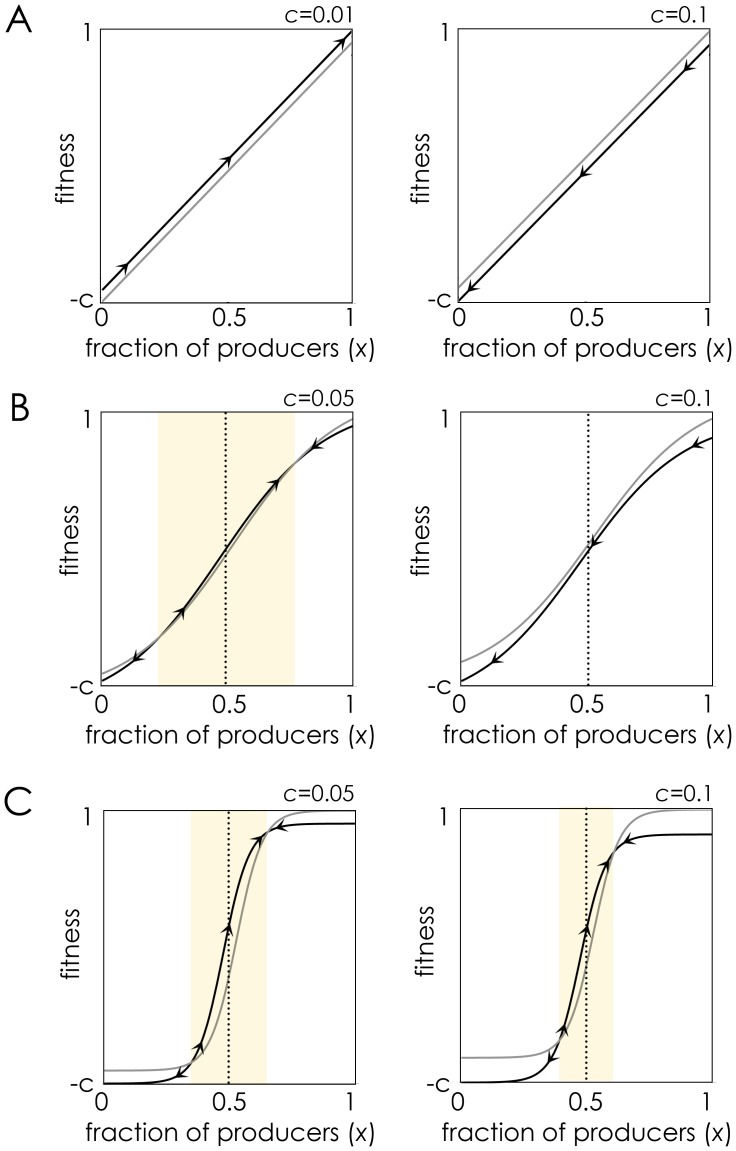
Why only non-linear benefits can lead to coexistence of producers and non-producers. The fitness of producers (black curve) and non producers (grey curve) as a function of the fraction (*x*) of producers within the diffusion range of the molecule, for different steepness coefficients (*s*). The arrows show the direction of the dynamics. The shaded area shows the basin of attraction of the internal stable equilibrium (if it exists). *n* = 20, *h* = 0.5. **A:** The benefit *B*(*x*) of the molecule is an almost linear (*s* = 0.001) function of its concentration. **B:** The benefit *B*(*x*) is a sigmoid (*s* = 5) function of its concentration. **C:** The benefit function is essentially a step function (*s* = 20).

The logic of non-linear benefits on the dynamics of public goods is explained in **Figure 4** (see also previous discussions for well-mixed populations [9], [18]: If the benefit of the molecule is a linear function of its concentration (s→0), either producers or non-producers have a higher fitness for any frequency of producers, depending only on the relative cost/benefit of producing the molecule (in sizeable groups, for reasonable costs producers will always have a disadvantage, this results in what we usually refer to as “N-person Prisoner’s Dilemma”, and hence to what is generally referred to as a “tragedy of the commons” [8]). If benefits are non-linear, however (larger *s*) and the cost *c* is not too high, a stable polymorphism is possible. Note that large *s* values (steep benefit functions) allow stable polymorphic equilibria for larger values of *c*, but they make the population less robust to random fluctuations in the fraction of producers, that is, to a smaller basin of attraction for the stable polymorphic equilibrium [**Figure 4**].

**Figure 5:**
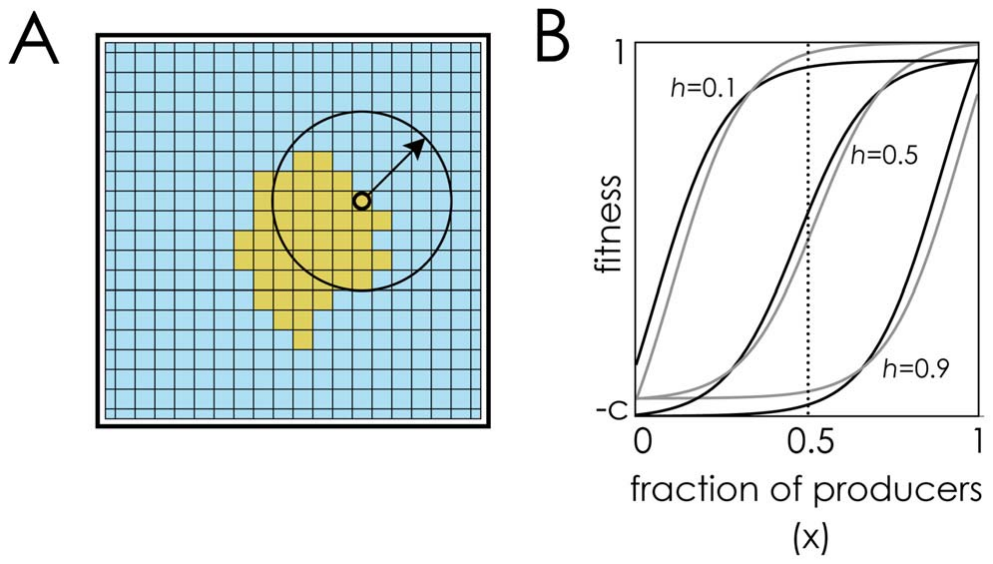
Why sigmoid benefits lead to different results from concave and convex benefits in spatially structured populations. The fraction of producers within the diffusion range (shown by an arrow) of molecules produced by cells at the producer/non-producer front remains approximately constant (∼0.5) even as the front moves ahead. If the benefit function is concave (*h* = 0.1) or convex (*h* = 0.9), at this fraction of producers (∼0.5) non-producers have an advantage, whereas producers have an advantage if the benefit function is sigmoid (*h* = 0.5). (*n* = 20, *c* = 0.1, *s* = 10).

The above argument is valid for well-mixed populations. In spatially structured populations the logic requires one further step. The crucial point is that, while in a well-mixed population the fraction of producers in the group is approximately the same as the frequency in the population (because new groups are formed at each generation), this is not the case in a spatially structured population, where the local fraction of producers can be much lower or higher than in the rest of the population. Consider a cluster of non-producers in a population of producers [**Figure 5A**]: the group defined by the diffusion range of the molecules produced by a cell at the edge between producers and non-producers is made by approximately half producers and half non-producers (if the diffusion range and the clusters are large enough); if non-producers have a higher fitness than producers (which is always the case if benefits are linear and *c* is not too small), the non-producer cluster will expand and the producer/non-producer front will move ahead; the process will go on with new groups (new producer/non-producer edges) until the whole population is made of non-producers. The same will happen when benefits are concave or convex, but not when benefits are sigmoid [**Figure 5B**]. If population structure is more complex (that is, if there is more than one cluster of non-producers in a population of producers) the intuition provided by Figure 5 fails, although it is clear that with concave or convex benefits (*h* close 0 or close to 1 in **Figure 2**) producers will ultimately go to extinction, whereas with sigmoid benefits the position of producer and non-producer cells will fluctuate around a mixed stable equilibrium [**Figure 1** **, Movie S1**].

## Discussion

### Producers and non-producers coexist if benefits are sigmoid

In summary, in a public goods game with two types of cells, producers and non-producers of a diffusible molecule, a stable polymorphism of the two types is likely under a wide range of parameters if the effect of the molecule is a sigmoid function of its concentration. The shape of the diffusion gradient is largely irrelevant and even a simple step function (that is, a model in which the diffusible molecule is fully functional up to a certain range and completely ineffective beyond that range) is accurate enough. These results, therefore, are similar to results in well-mixed populations [9], where coexistence of producers and non-producers is commonly observed. In short, the difference between Borenstein et al. [13] and Archetti [15] is the shape of the benefit function, not the shape of the diffusion gradient. The conclusion of Borenstein et al. [13] that coexistence is impossible depends on their assumption that benefits are linear.

Borenstein et al. [13] conclude that their assumption of linearity does not affect their result, and that non-linear benefits would lead to the same result (no coexistence of the two types). Their discussion of non-linearities, however, assumes a concave benefit function, rather than a sigmoid function. The extinction of one of the two types when benefits are concave is also observed in a model without diffusion gradient [15]. Scheuring [14] observes coexistence with concave benefits in a 1-D model. Here we have seen that concave benefits can actually lead to coexistence of the two types only under very restrictive conditions (the production cost must not be too high). Coexistence of producers and non-producers, however, is the typical result of the dynamics if benefits are sigmoid.

### Sigmoid benefits are common in biological public goods

The relevance of these results depends on how common linear, sigmoid or concave benefits are in nature. Borenstein et al. [13] ask “could a saturating resource uptake curve, such as the Michaelis-Menten model facilitate coexistence?” (concluding that the answer is no). The Michaelis-Menten equation discussed by Borenstein et al. [13], however, is a concave function. Biological input-output systems, instead, generally follow the Hill equation [19], [20], that is, biological input-output systems generally show a slow response at low inputs levels followed by a steep increase in response at intermediate levels and again a decreasing sensitivity as input levels increases. In other words, the effect of a biological molecule is often a sigmoid, not concave, function of its concentration [20]–[23]. Examples of sigmoid benefits have been reported for public goods in both microbes [24], and cancer cells [25].

There are various proximate explanations for the occurrence of sigmoid benefits in nature. The most basic explanation [20] is *cooperative binding*: transforming a single molecule to an active state may require simultaneous binding by multiple input signal molecules. Other explanations [23] include *titration of a repressor* (the initial reaction may inactivate the input signal molecule or reduce sensitivity to low intensity input signals), and *opposing saturated forward and back reactions* (a back reaction may return the active form produced by the initial reaction to the inactive state, and if the back reaction saturates at low signal input intensity, then a logarithmic output will result at low input intensity). More in general, the reason for the common occurrence of “Hill kinetics” is that the final physiological or behavioral response of a biological system is produced by a cascade of signal originating from cellular receptors and sensory systems. This series of reactions amplifies even the slightest departure from linearity of the underlying individual chemical reactions [21].

Since biological molecules generally follow the Hill kinetics (sigmoid benefits) rather than the Michaelis-Menten kinetics (concave benefits), and because linear benefits are unlikely to exist at all in nature, the coexistence of producers and non-producers is likely to be the typical outcome of cooperation for the production of diffusible molecules.

### Further work on diffusible public goods

While, as we have seen, the exact shape of the diffusion gradient is not essential, the diffusion range of the molecule is important in determining the dynamics of the production of diffusible molecules. Here we have assumed that the diffusion range is small, thus group size (the interaction neighborhood) is always one order of magnitude smaller than population size. If the diffusion range is such that group size approaches population size, however, the extinction of one of the two types becomes possible [10], [17]. Measuring the diffusion range of molecules that act as public goods is therefore important to understand their dynamics.

While Borenstein et al. [13] use a stochastic update rule, both Scheuring [14] and Archetti [15] show that whether update is deterministic or stochastic does not lead to substantially different results, except for the time required to reach a polymorphism. The details of the update rule, however, may be important. Scheuring [14] shows that, in his 1-D model, concave benefits lead to coexistence when using the birth-death update rule (the same rule used by both Borenstein et al. [13], Archetti [15] and here), but not with a death-birth rule. It is understood that very different results can be expected in spatially structured populations based on whether local and global selection are random or proportional to fitness (a more appropriate classification of update rules than the mere order of birth and death [26]).

The combined results of Borenstein et al. [13], Scheuring [14], Archetti [15], and the ones reported here help us understand the importance of non-linear benefits and of diffusion gradients; Scheuring [14] goes one step further and analyses a further update rule, although in a 1-D model and only with linear and concave benefits; Allen et al. [16] make a thorough analysis of diffusion and use different update rules, but their analysis is limited to linear benefits. It would be worth extending these studies of diffusible public goods to analyse the effect of different update rules on 2-D games with different update rules and benefits functions.

## Conclusion

As noted recently by Borenstein et al. [13], Scheuring [14], Archetti [15] and Allen et al. [16], the dynamics of public goods production in biological systems must be analysed by games in which the interaction neighbourhood extends beyond the update neighbourhood. As we have seen here, however, the precise shape of the diffusion gradient has a relatively little impact on the results, and therefore an accurate treatment of diffusion is not essential; a simple diffusion range without decreasing efficacy is a good enough approximation. The crucial assumption of the models, instead, is the shape of the benefit function. Since the dynamics of non-linear public goods is so radically different from the dynamics with linear benefits, and since biological public goods are generally non-linear, the production of diffusible molecules in biological systems cannot be reliably approximated by models with linear benefits, even though the analysis of non-linear games is by far more complex and rarely amenable to analytical proofs. In a model with non-linear benefits, and therefore in a population of cells producing a diffusible molecule, stable polymorphism is not only possible, but the likely outcome of the dynamics.

## Supporting Information

Movie S1 **Long-term coexistence of producers and non-producers.** Population structure (blue: producer cells; yellow: non-producer cells) and fitness, from -*c* (grey) to 1 (pink) (as in Figure 1) during the first 200 divisions per cell, after a cluster of non-produce cells arises in a population of producers; sigmoid benefit (*s* = 20, *h* = 0.5), no diffusion gradient (*z* = 1000, *d* = 3, *D* = 6), *c* = 0.05.
(MOV)

## References

[1] Crespi BJ (2001) The evolution of social behavior in microorganisms. Trends Ecol. Evol. 16: 178–183. 11245940 10.1016/s0169-5347(01)02115-2

[2] Wang Y, Kern SE, Newman DK (2010) Endogenous phenazine antibiotics promote anaerobic survival of Pseudomonas aeruginosa via extracellular electron transfer. Journal of Bacteriology 192: 365–369. 19880596 10.1128/JB.01188-09 PMC2798253

[3] Meyer JM (2000) Pyoverdines: pigments, siderophores and potential taxonomic markers of fuorescent Pseudomonas species. Archives of Microbiology 174: 135–142. 11041343 10.1007/s002030000188

[4] Rainey PB, Rainey K (2003) Evolution of cooperation and conflict in experimental bacterial populations. Nature 425: 72–74. 12955142 10.1038/nature01906

[5] Lee HH, Molla MN, Cantor CR, Collins JJ (2010) Bacterial charity work leads to population-wide resistance. Nature 467: 82–86. 20811456 10.1038/nature09354 PMC2936489

[6] Greig D, Travisano M (2004) The prisoner’s dilemma and polymorphism in yeast SUC genes. Proc. Roy. Soc. B 271: S25–S26. 10.1098/rsbl.2003.0083 PMC1810003 15101409

[7] Axelrod R, Axelrod DE, Pienta KJ (2006) Evolution of cooperation among tumor cells. Proc. Natl. Acad. Sci. USA 103: 13474–13479. 16938860 10.1073/pnas.0606053103 PMC1557388

[8] Hardin J (1968) The tragedy of the commons. Science 162: 1243–1248. 5699198

[9] Archetti M, Scheuring I (2012) Review: Game theory of public goods in one-shot social dilemmas without assortment. J. Theor. Biol. 299: 9–20. 21723299 10.1016/j.jtbi.2011.06.018

[10] Perc M, Gómez-Gardeñes J, Szolnoki A, Floria LM, Moreno Y (2013) Evolutionary dynamics of group interactions in structured populations: A review. J. R. Soc. Interface 10: 20120997. 23303223 10.1098/rsif.2012.0997 PMC3565747

[11] Ifti M, Killingback T, Doebeli M (2004) Effects of neighbourhood size and connectivity on the spatial continuous prisoner’s dilemma. J. Theor. Biol. 231: 97–106. 15363932 10.1016/j.jtbi.2004.06.003

[12] Ohtsuki H, Pacheco JM, Nowak MA (2007) Evolutionary graph theory: breaking the symmetry between interaction and replacement. J. Theor. Biol. 246: 681–694. 17350049 10.1016/j.jtbi.2007.01.024 PMC2396517

[13] Borenstein DB, Meir Y, Shaevitz J, Wingreen NS (2013) Non-local interaction vie diffusible resource prevents coexistence of cooperators and cheaters in a lattice model. PLOS One 8: e63304. 23691017 10.1371/journal.pone.0063304 PMC3656920

[14] Scheuring I (2013) Diffusive public goods and coexistence of cooperators and cheaters on a 1D lattice. PLOS One 9(7): e100769. 10.1371/journal.pone.0100769 PMC4098918 25025985

[15] Archetti M (2013) Dynamics of growth factor production in monolayers of cancer cells and evolution of resistance to anticancer therapies. Evolutionary Applications 6: 1146–1159. 24478797 10.1111/eva.12092 PMC3901545

[16] Allen B, Gore J, Nowak MA (2013) Spatial dilemmas of diffusible public goods. eLife 2: e01169. 24347543 10.7554/eLife.01169 PMC3865686

[17] Antal T, Scheuring I (2006) Fixation of strategies for an evolutionary game in finite populations. Bulletin of Mathematical Biology 68: 1923–1944. 17086490 10.1007/s11538-006-9061-4

[18] Archetti M, Scheuring I (2011) Coexistence of cooperation and defection in public goods games. Evolution 65: 1140–1148. 21062277 10.1111/j.1558-5646.2010.01185.x

[19] Hill AV (1910). The possible effects of the aggregation of the molecules of hæmoglobin on its dissociation curves. Proceedings of the Physiological Society, Jan 1910.

[20] Cornish-Bowden A (2012) *Fundamentals of Enzyme Kinetics*, 4th edition; Wiley Blackwel.

[21] Frank, S. A. Input-output relations in biological systems: measurement, information and the Hill equation. Biology Direct 8: 31. 24308849 10.1186/1745-6150-8-31 PMC4028817

[22] Tyson JJ, Chen KC, Novak B (2003) Sniffers, buzzers, toggles and blinkers: dynamics of regulatory and signaling pathways in the cell. Curr Opin Cell Biol 15: 221–231. 12648679 10.1016/s0955-0674(03)00017-6

[23] Zhang Q, Bhattacharya S, Andersen ME (2013) Ultrasensitive response motifs: basic amplifiers in molecular signalling networks. Open Biol 3: 130031. 23615029 10.1098/rsob.130031 PMC3718334

[24] Chuang JS, Rivoire O, Leibler S (2010) Cooperation and Hamilton’s rule in a simple synthetic microbial system. Mol Syst Biol. 6: 398. 20706208 10.1038/msb.2010.57 PMC2950083

[25] Jourdan M, Mahtouk K, Veyrune J, Couderc G, Fiol G, et al (2005) Delineation of the roles of paracrine and autocrine interleukin-6 (IL-6) in myeloma cell lines in survival versus cell cycle. A possible model for the cooperation of myeloma cell growth factors. Eur. Cytokine Netw. 16: 57–64. 15809207

[26] Grafen A, Archetti M (2008) Natural selection of altruism in inelastic viscous homogeneous populations. J. Theor. Biol. 252: 694–710. 18371985 10.1016/j.jtbi.2008.01.021

